# The function of VAMP2 in mediating membrane fusion: An overview

**DOI:** 10.3389/fnmol.2022.948160

**Published:** 2022-12-23

**Authors:** Chong Yan, Jie Jiang, Yuan Yang, Xiaoqi Geng, Wei Dong

**Affiliations:** ^1^Key Laboratory of Medical Electrophysiology, Ministry of Education and Medical Electrophysiological Key Laboratory of Sichuan Province, Institute of Cardiovascular Research, Southwest Medical University, Luzhou, Sichuan, China; ^2^Department of Neurosurgery, Neurosurgical Clinical Research Center of Sichuan Province, Affiliated Hospital of Southwest Medical University, Luzhou, China

**Keywords:** VAMP2, SNAREs, juxta membrane, membrane fusion, synaptic vesicle

## Abstract

Vesicle-associated membrane protein 2 (VAMP2, also known as synaptobrevin-2), encoded by VAMP2 in humans, is a key component of the soluble *N*-ethylmaleimide-sensitive factor attachment protein receptor (SNARE) complex. VAMP2 combined with syntaxin-1A (SYX-1A) and synaptosome-associated protein 25 (SNAP-25) produces a force that induces the formation of fusion pores, thereby mediating the fusion of synaptic vesicles and the release of neurotransmitters. VAMP2 is largely unstructured in the absence of interaction partners. Upon interaction with other SNAREs, the structure of VAMP2 stabilizes, resulting in the formation of four structural domains. In this review, we highlight the current knowledge of the roles of the VAMP2 domains and the interaction between VAMP2 and various fusion-related proteins in the presynaptic cytoplasm during the fusion process. Our summary will contribute to a better understanding of the roles of the VAMP2 protein in membrane fusion.

## 1 Introduction

The transmission of information between neurons in the central nervous system (CNS) relies primarily on the release of neurotransmitters at the synapse (Sudhof, 2008; Vardjan et al., 2012). Membrane fusion is a pivotal step in synaptic transmission, serving to ensure that synaptic vesicles (SVs) containing various neurotransmitters can be released as quickly as possible (on a millisecond time scale) upon the arrival of an action potential (Diao et al., 2012a; Brunger et al., 2015). Most SVs are released via Ca^2+^-dependent evoked release, although some fuse in a stimulus-independent mode known as spontaneous release (Sauvola and Littleton, 2021). The soluble *N*-ethylmaleimide-sensitive factor attachment protein receptor (SNARE) complex, which is formed in the active zone (AZ) of the presynaptic cytoplasm, has been demonstrated to play a key role in membrane fusion (Tian et al., 2019; Hesselbarth and Schmidt, 2021). The components of the SNARE complex, which assemble into a parallel four-helix bundle, are generally divided into two categories based on their localization, namely, v-SNAREs (located on vesicles) and t-SNAREs (located on target membrane) (Hong, 2005; Tian et al., 2019). v-SNAREs, also designated as vesicle-associated membrane proteins (VAMPs), and t-SNAREs, including syntaxin-1A (SYX-1A) and synaptosomal-associated protein 25 (SNAP-25), together constitute the minimum fusion mechanism required for the preparation of SVs for fusion as well as for the fusion between SVs and the plasma membrane in neuronal cells (Sollner et al., 1993; Weber et al., 1998; Deák et al., 2006; Rizo and Rosenmund, 2008; Brunger et al., 2015; Neher and Brose, 2018; Tian et al., 2019).

The v-SNARE comprises a VAMPs family of proteins that are present on the surface of SVs at nerve terminals (Raptis et al., 2005). Important homologous VAMP isoforms include VAMP1, VAMP2, VAMP3, VAMP4, VAMP7, and VAMP8 (Table 1; Elferink et al., 1989; Rossi et al., 2004; Hong, 2005; Scheuber et al., 2006; Meunier et al., 2010; Ramirez et al., 2012; Malmersjö et al., 2016; Ivanova et al., 2021; Tian et al., 2021; Ivanova and Cousin, 2022). In mammalian cells, these six VAMPs reside in a variety of post-Golgi vesicular compartments and mediate vesicle fusion with the plasma membrane, the trans-Golgi network, and endosomes (Meunier et al., 2010). In addition, it has been reported that multiple v-SNAREs support spontaneous and asynchronous SV release in mammalian synapses (Ramirez et al., 2012; Chung and Raingo, 2013; Lin et al., 2020). VAMP1 and VAMP2 are involved in regulated exocytosis in neurons and endocrine cells (Kesavan et al., 2007; Meunier et al., 2010; Hua et al., 2011; Shimojo et al., 2015). VAMP1 was proposed to mediate vesicle priming and evoked release in subpopulations of hippocampal neurons. In a culture system of mouse hippocampal VAMP2 knockout neurons, in which spontaneous neurotransmission still occurs, the spontaneous release activity of a small group of neurons was reported to be correlated with the expression of VAMP1. Moreover, the authors performed VAMP1 rescue experiments in VAMP2-deficient neurons and found that VAMP1 can substitute for the loss of VAMP2 in the maintenance of neurotransmitter release in central synapses. However, VAMP1 exhibited lower efficiency in promoting evoked and spontaneous release compared with VAMP2 (Deák et al., 2004; Zimmermann et al., 2014). VAMP1 also regulates Ca^2+^-induced neurotransmitter release at the mouse neuromuscular junction (NMJ). Liu et al. (2011) examined NMJ function in VAMP1 mutant mice and found that the loss of VAMP1 did not impair the formation of muscle synapses but instead led to a reduction in spontaneous synaptic activity and significant reductions in evoked synaptic transmission and initial release probability. These results indicated that VAMP1 is essential for the maintenance of nerve impulse transmission in neuromuscular synapses (Pang and Sudhof, 2010; Liu et al., 2011). VAMP8 was the first member of the VAMP family to be discovered, and its role in exocytosis and vesicle transport has been confirmed (Behrendorff et al., 2011; Messenger et al., 2014; Wang et al., 2018). However, it was recently reported that VAMP8, the SNARE protein on lysosomes, is strongly associated with the SNARE-mediated fusion of lysosomal and autophagosomal membranes in autophagy (Chen et al., 2021). VAMP3, VAMP4, and VAMP7 also have selective roles in vesicle trafficking events, as listed in Table 1.

**TABLE 1:** The role of VAMPs family in vesicle fusion.

VAMP	Functions in vesicle fusion	References
VAMP1	VAMP1 mediates vesicle priming and evoked release in a subpopulation of hippocampal neurons	Zimmermann et al., 2014
VAMP2	Tetanus-insensitive VAMP2 differentially restores synaptic and dense core vesicle fusion in tetanus neurotoxin-treated neurons	Hoogstraaten et al., 2020
	Mutations in the neuronal vesicular SNARE VAMP2 affect synaptic membrane fusion and impair human neurodevelopment	Salpietro et al., 2019
	A central small amino acid in the VAMP2 transmembrane domain regulates fusion pore opening and expansion in exocytosis	Hastoy et al., 2017
	The interaction of helices 11 and 12 of Munc18-1 with the central region of the VAMP2 SNARE motif is essential for SNARE templating and synaptic transmission	Andre et al., 2020
	VAMP2 transmembrane domain (TMD) residues influence the kinetics of synaptic release. TMD mutations alter the max-rise rate of miniature excitatory postsynaptic currents (mEPSCs) in a manner consistent with interactions with glutamate as it passes through the fusion pore The diffusion characteristics of VAMP2 and SYT proteins may influence the rate of exocytosis The membrane-proximal regions of VAMP2 and SNAP25 can be captured by complexin, leading to the suppression of spontaneous exocytosis Electrostatic interactions between VAMP2 and acidic phospholipids may modulate the fusion of transport vesicles with the plasma membrane α-Synuclein may cross-bridge VAMP2 and acidic phospholipids to facilitate SNARE-dependent vesicle docking	Chiang et al., 2018 Abbineni et al., 2022 Malsam et al., 2020 Williams et al., 2009 Lou et al., 2017
	VAMP2 mediates vesicle fusion with the plasma membrane and is involved in the trafficking of alpha-5 beta-1 integrin	Hasan and Hu, 2010
	VAMP2-dependent regulation of single synaptic vesicle endocytosis	Chanaday and Kavalali, 2021
	Two VAMP2 molecules and likely two SNARE complexes are necessary and sufficient for synaptic vesicle (SV) fusion during fast synaptic transmission	Sinha et al., 2011
	A large vesicular VAMP2 pool maintained by AP180 is crucial for sustaining efficient neurotransmission and SV reformation	Koo et al., 2015
	The VAMP2 TMD plays an important role in nascent fusion pores	Chang et al., 2015
	The VAMP2 TMD catalyzes the fusion process through its structural flexibility, actively setting the pace of fusion pore expansion	Dhara et al., 2016
	VAMP2 can be bound by native α-synuclein to induce the clustering of synaptic-vesicle mimics	Diao et al., 2013
	The tail domain of tomosyn controls membrane fusion by serving as a placeholder for VAMP2	Yamamoto et al., 2010
	VAMP2 TMD-mediated support of membrane curvature and SNARE force-generated membrane bending promote fusion pore formation and expansion	Dhara et al., 2020
	VAMP2 is the v-SNARE required for cytotoxic T-lymphocyte lytic granule fusion	Matti et al., 2013
	VAMP2 contributes to both regulated and constitutive AMPAR exocytosis	Jurado et al., 2013
	Syntaxin4/SNAP-23/VAMP2 negatively regulate Munc18c-mediated membrane fusion *in vitro*	Brandie et al., 2008
	Juxtamembrane tryptophans of VAMP2 control the process of membrane fusion	Fang et al., 2013
	Three to four copies of VAMP2 (a minimum of two per face) are required to keep a nascent fusion pore open; SNARE proteins act cooperatively to dilate the nascent fusion pore	Bello et al., 2016
	Optimum flexibility and membrane binding of VAMP2 regulate SNARE assembly and minimize repulsive forces during membrane fusion	Lakomek et al., 2019
	Synaptophysin mediates the efficient retrieval of VAMP2, thereby sustaining neurotransmitter release	Kokotos et al., 2019
	The C-terminal half of the VAMP2 TMD is essential for SNARE-mediated membrane-fusion events in cells	Fdez et al., 2010
	Complexin 2 modulates VAMP2-regulated zymogen granule exocytosis in pancreatic acini	Falkowski et al., 2010
	VAMP2 mediates Ca^2+^-triggered exocytosis by the tight coupling of the SNARE motif to the transmembrane region, hence forcing the membranes into close proximity for fusion	Deák et al., 2006
	SNAP23/25 and VAMP2 mediate the exocytosis of transferrin receptor-containing recycling vesicles	Kubo et al., 2015
	A direct interaction between Cdc42 and VAMP2 regulates SNARE-dependent insulin exocytosis	Nevins and Thurmond, 2005
VAMP3	VAMP3/SYB and YKT6 are required for the fusion of constitutive secretory carriers with the plasma membrane	Gordon et al., 2017
	The STX6-VTI1B-VAMP3 complex facilitates xenophagy by regulating the fusion between recycling endosomes and autophagosomes	Nozawa et al., 2017
	Transport vesicles containing VAMP3 have distinct membrane fusion kinetics with domains of the plasma membrane that present different t-SNARE proteins VAMP3 is associated with endothelial Weibel-Palade bodies and participates in their Ca^2+^-dependent exocytosis	Hu et al., 2007 Pulido et al., 2011
VAMP4	VAMP4 maintains a Ca^2+^-sensitive pool of spontaneously recycling synaptic vesicles	Lin et al., 2020
	The targeting of VAMP4 to endolysosomes regulates synaptic vesicle release probability	Ivanova et al., 2021
	VAMP4 directs synaptic vesicles to a pool that selectively maintains asynchronous neurotransmission	Raingo et al., 2012
VAMP7	VAMP7 mediates autophagosome-lysosome fusion VAMP7 is involved in the regulation of asynchronous and spontaneous release at the moss fiber terminals.	Scheuber et al., 2006; Aoyagi et al., 2018; Tian et al., 2020, Tian et al., 2021
VAMP8	VAMP8 mediates autophagosome-lysosome fusion	Tian et al., 2021

**FIGURE 1:**
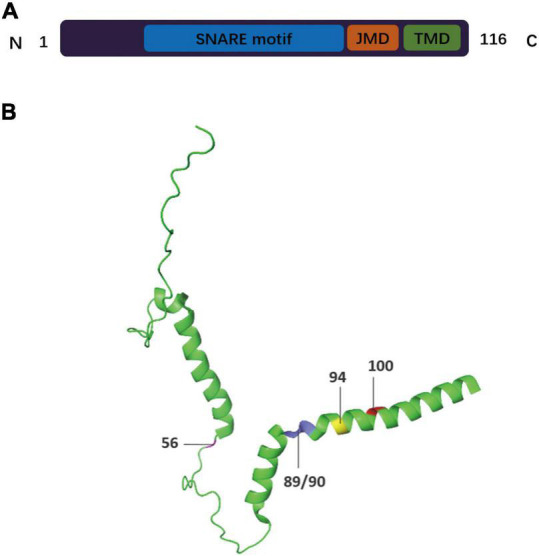
Schematic drawing of the domain arrangement and 3-D structure of VAMP2. **(A)** The primary structure of VAMP2 is displayed in bar format. The full-length protein of VAMP2 is characterized by (i) a 94 residues-long cytosolic domain including the highly conserved SNARE motifs (residues 31–85, blue) and the dynamic juxtamembrane domain (JMD, residues 86–94, orange), (ii) a transmembrane domain (TMD, residues 95–114, green) anchoring the protein to the vesicular membrane, and (iii) a short luminal domain of only 2 residues (residues 115/116). **(B)** The 3D structure of VAMP2 (Protein Data Bank ID: 2KOG). The residue sites discussed in this review are denoted as follows: residue 56 Arginine (magenta), residues 89/90 Tryptophan (blue), residue 94 Lysine (yellow), residue 100 Glycine (red).

VAMP2, also known as synaptobrevin-2 (SYB2), is a key SV fusogenic protein. SVs are densely packed with a wide variety of proteins that play important roles in the SV cycle (Hesselbarth and Schmidt, 2021). VAMP2 is the most abundant and widely distributed SV protein in the brain, with up to 70 copies per SV (Südhof et al., 1989; Takamori et al., 2006; Südhof and Rothman, 2009; Liu et al., 2011; Ahnert-Hilger et al., 2013; Mori and Takamori, 2017; Wittig et al., 2021). VAMP2 has a well-characterized and conserved role in synaptic function and has been demonstrated to be mainly involved in the assembly of effective SNARE complexes, Ca^2+^-dependent SV exocytosis, and fast endocytosis in hippocampal synapses (Schoch et al., 2001; Deák et al., 2004; Raptis et al., 2005; Südhof and Rothman, 2009; Rizo and Südhof, 2012; Orock et al., 2020). VAMP2 is the main VAMP isoform present in neuronal large dense core vesicles (DCVs) that facilitate the release of catecholamine. One study showed that inhibiting the expression of VAMP2 in cortical neurons using short hairpin RNA can reduce the exocytosis of DCVs, including those that release brain-derived neurotrophic factor (BDNF) (Shimojo et al., 2015). Other studies have also explored the role of VAMP2 in DCV exocytosis in tetanus neurotoxin (TeNT)-treated hippocampal neurons and demonstrated that VAMP2 is the only known v-SNARE that can support DCV fusion (Regazzi et al., 1995; Janz et al., 1999; Sinha et al., 2011; Persoon et al., 2018; Bulgari et al., 2019; Fezoua-Boubegtiten et al., 2019; Hoogstraaten et al., 2020). However, it is unclear whether VAMP2 mediates all DCV fusion events in the brain. VAMP2 also participates in neurotransmitter release during neuronal exocytosis by actively maintaining its own position and levels on the vesicle membrane and interacting with other SNAREs and related proteins (Raptis et al., 2005; Gordon and Cousin, 2014; Han et al., 2016; Weiss, 2019). VAMP2 is largely unstructured and has disordered filaments in its native state (Fasshauer, 2003). Upon interacting with other SNAREs, the VAMP2 transformed to four structural domains, including three disordered soluble domains—a proline-rich and relatively short N-terminal domain (residues 1–30); a SNARE motif, also known as the core domain (residues 31–85); a juxtamembrane domain (JMD, residues 86–95)—and a single C-terminal transmembrane domain (TMD, residues 96–114) (Figure 1; Deák et al., 2006; Rizo and Südhof, 2012; Diao et al., 2013; Fang et al., 2013; Wang C. et al., 2020). The transformation from a disordered to an ordered state may release free energy to drive the transformation of SNARE complex, which indicates the conformational diversity of synaptic SNARE proteins (McNew et al., 2000; Hesselbarth and Schmidt, 2021; Sauvola and Littleton, 2021). The TMD serves as the scaffold for the anchoring of VAMP2 to the vesicle membrane (Deák et al., 2006; Fang et al., 2013), while the other three domains are located outside the vesicle (Wang C. et al., 2020). Several studies have shown that the TMD of VAMP2 is vital for the fusion process, from its initiation to the opening of the fusion pore (Han et al., 2004, 2016; Xu et al., 2005; Deák et al., 2006; Kesavan et al., 2007; Bretou et al., 2008; Fdez et al., 2010; Cai et al., 2020), while the JMD is also essential for vesicle fusion (Rathore et al., 2019). This suggests that the different domains of VAMP2 have complex roles in vesicle/membrane fusion events during vesicle endocytosis and neurotransmitter release. In this review, we mainly elucidate the functional roles of VAMP2 in membrane fusion reactions and the characteristics of the different structural domains of VAMP2 after the formation of the catalytic SNARE complex. We also discuss how VAMP2 interacts with other proteins during the membrane fusion process.

## 2 The role of VAMP2 in membrane fusion

**FIGURE 2:**
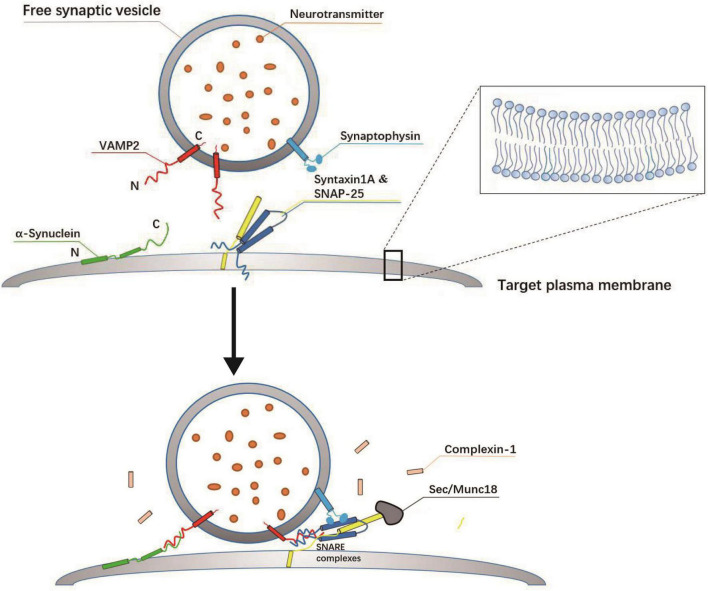
Assembly mechanism of SNARE complex at the target plasma membrane. SNARE complex proteins are displayed as cylinder icons. The soluble *N*-ethylmaleimide sensitive factor attachment receptor (SNARE): SYX-1A (yellow), SNAP-25 (dark blue), and VAMP2 (red) form a four-helix cross SNARE complex (one from VAMP2, one from SYX-1A, and two from SNAP-25), connecting vesicles and plasma membrane, which is the key to membrane fusion. The SM protein (gray) activates the formation of the SNARE zipper, which brings the vesicle membrane and the target membrane close to each other, so the membrane fusion is thought to be the result of the synergistic action of Munc-18-1 and SNARE. In addition, synaptophysin (light blue) is thought to control the transport of VAMP2 after fusion, also located on the vesicle membrane. The complexin (pink) is a key regulator of the core fusion machinery and is required for vesicle fusion and exocytosis.

Membrane fusion refers to the merging of two lipid bilayers into one through membrane remodeling and lipid rearrangements (Martens et al., 2007; Südhof and Rothman, 2009; Kozlov et al., 2010; Diao et al., 2011; Brunger et al., 2015). A well-characterized form of membrane fusion is the fusion of intracellular vesicles with target membranes involved in the transport of cargo proteins between intimal organelles (Hu et al., 2007; Ohya et al., 2009; Wickner, 2010; Diao et al., 2012b; Baker et al., 2015). The SNARE complex is considered to be a bridge between intracellular vesicles and the plasma membrane in addition to being a core fusion element (Shi et al., 2012; Fang and Lindau, 2014; Brunger et al., 2015; Tian et al., 2019). Binding and assembly of SNARE proteins are sufficient to drive the fusion of SVs (McNew et al., 2000; Li et al., 2020); however, the fast kinetics of membrane fusion, which occurs in the order of milliseconds, requires the precise coordination of SNAREs and auxiliary synaptic proteins (Weber et al., 1998; Rizo and Rosenmund, 2008; Zhou et al., 2017; Neher and Brose, 2018; Salpietro et al., 2019; Tian et al., 2019; Li et al., 2020; Sauvola and Littleton, 2021). The key regulatory components include chaperones Sec1/Munc18 (SM) protein family, the primary Ca^2+^ sensor synaptotagmin (SYT), complexins (CPX) protein, and *N*-ethylmaleimide sensitive factor (NSF) (Weber et al., 1998; Melia et al., 2002; Rizo and Rosenmund, 2008; Südhof and Rothman, 2009; Diao et al., 2011; He et al., 2017; Zhou et al., 2017; Salpietro et al., 2019; Li et al., 2020; Wang C. et al., 2020; Wang X. et al., 2020). Membrane fusion is activated when SNAREs and SM proteins facilitate the coming together of vesicle and target membranes (Figure 2; Xu et al., 2010; Jiao et al., 2018; Rathore et al., 2019). SM proteins ensure the homology and specificity of SNARE pairing as well as vesicle fusion with the correct target membrane (De Haro et al., 2003; Shen et al., 2007; Xu et al., 2010; Ma et al., 2013; Baker et al., 2015; Rizo, 2018; Yu et al., 2018; Sauvola and Littleton, 2021). CPX protein specifically regulates the fusion mechanism that promotes the loading of SVs in the AZ and is involved in exocytosis and the recycling of vesicles in the cytosol (Figure 2; Tang et al., 2006; Zhou et al., 2017; Scholz et al., 2019). These proteins, each representing a small protein family, cooperate to provide rapid, effective, and specific fusion, thereby preventing the formation of unproductive intermediates (Rathore et al., 2019; Sauvola and Littleton, 2021).

The importance of VAMP2 in SV/membrane fusion has been demonstrated using a variety of research methods. The electrostatic interaction between VAMP2 and SYX-1A induces membrane bending forces, which results in the pulling together of SVs and presynaptic membranes, thereby facilitating membrane fusion and exocytosis events (Williams et al., 2009; Gundersen, 2017; Hesselbarth and Schmidt, 2021). Wittig et al. (2021) applied cross-linking mass spectrometry to study the interactions among SV proteins using purified, unstimulated SVs. Strikingly, the resulting interaction network showed that VAMP2 was cross-linked with 32 of the 56 proteins assayed. These results supported a key role for VAMP2 in SNARE complex formation (Wittig et al., 2021). Meanwhile, X-ray crystallography-based studies indicated that the SNARE complex continues to extend until the helix almost reaches the TMDs of VAMP2 and SYX-1A post-fusion, such that the vesicle and the plasma membranes are pulled very close together (Fang et al., 2008; Stein et al., 2009). In this scenario, to achieve fusion, the bilayer lipids would have to be disrupted, and the conformation of the SNARE complex would have to change. These changes would thus provide a force to overcome the energy barrier of membrane fusion, and the force generated by the composite SNARE zipper would alter the position and direction of the VAMP2 TMD (Bretou et al., 2008; Fang et al., 2008; Ngatchou et al., 2010). Mutations of residues in the TMD of VAMP2 can affect membrane fusion. For instance, they can disrupt the interaction between VAMP2 and t-SNAREs, resulting in a reduction in active fusion vesicle numbers. Meanwhile, fusion pore flux, such as the exocytosis of catecholamine-containing granules, is sensitive to the structural flexibility of TMDs. For instance, when the core residues (amino acids 97–112) of the VAMP2 TMD were changed to leucine, thereby imparting greater helical stability and less flexibility, exocytosis was significantly reduced, and fusion pore opening was slower in both hippocampal neurons and a type of neuroendocrine cell known as a chromaffin cell. In contrast, when the core residues were changed to helix-destabilizing, β-branched isoleucine or valine residues, exocytosis occurred normally, although the fusion pore expanded faster when compared with that in the wild-type condition (Risselada et al., 2011; Chang and Jackson, 2015; Bao et al., 2016; Dhara et al., 2016). In summary, it seems likely that the VAMP2 TMD and t-SNAREs jointly induce fusion initiation and fusion pore expansion.

Vesicle-associated membrane protein 2 mutations inhibit neurotransmitter release. Studies employing electrophysiological recordings demonstrated that VAMP2 knockdown significantly diminishes the frequency of spontaneous neurotransmitter release as monitored by miniature excitatory postsynaptic currents (mEPSCs) and miniature inhibitory postsynaptic currents (mIPSCs); however, the mEPSC and mIPSC amplitudes remained unchanged. Wild-type VAMP2 rescued the knockdown phenotype, whereas VAMP2 mutants did not. Importantly, VAMP2 depletion led to a strong reduction in the amplitudes of eEPSCs and eIPSCs evoked by local electrical stimulation in cultured neurons. These findings indicate that the knockdown of VAMP2 leads to a reduction in excitatory or inhibitory synapse transmission. Similarly, these defects were fully rescued by wild-type VAMP2 but not by VAMP2 mutants (Shen et al., 2015). In addition, a strong reduction in the readily releasable pool of vesicles (RRP), the synaptic vesicles that are immediately available for release, was also observed in VAMP2 knockdown cells, an effect that was rescued by WT VAMP2 but not mutant VAMP2. Most VAMP2 deficient neurons lacked Ca^2+^-mediated evoked release or spontaneous release and displayed no measurable RRP (Schoch et al., 2001; Zimmermann et al., 2014; Kokotos et al., 2019). Together, these results demonstrate that VAMP2 mutations strongly inhibit both spontaneous and evoked neurotransmitter release in cultured neurons. They also imply that VAMP2 plays a considerable role in specific membrane transport responses, including vesicle fusion, neurotransmitter release, and vesicle endocytosis (Schoch et al., 2001; Deák et al., 2004; Brunger et al., 2018; Kokotos et al., 2019).

## 3 Interaction between VAMP2 and other proteins

Vesicle-associated membrane protein 2 exists in the presynaptic cytoplasm at high concentrations (Lvov et al., 2008; Koo et al., 2015). Over the past few years, several proteins have been identified that interact with VAMP2 and exert critical functions in facilitating the assembly of SNARE complexes and the movement of key vesicle fusion-related molecules (Gordon and Cousin, 2014). These proteins include α-synuclein (α-Syn), synaptophysin (SYP), SM proteins, and some ion channel proteins (Pennuto et al., 2003; Lvov et al., 2008; Russell et al., 2012; Diao et al., 2013; Kubo et al., 2015; Lou et al., 2017; Andre et al., 2020). It has also been suggested that electrostatic interactions between VAMP2 and acidic phospholipids may regulate the fusion of transport vesicles with the plasma membrane (Williams et al., 2009).

### 3.1 Interaction of VAMP2 with α-syn

α-Syn is a cytoplasmic regulator of neurotransmission that associates with the neuronal extracellular secretory pathway and has been strongly implicated in several neurodegenerative diseases (Wong and Krainc, 2017; Sun et al., 2019). α-Syn is known to interact with negatively charged phospholipids and VAMP2 (Lotharius and Brundin, 2002; Burré et al., 2010; Lou et al., 2017; Cai et al., 2020; Figure 2). The C-terminal domain of α-Syn has been demonstrated to interact with the N-terminal amino acids of VAMP2 *in vitro*, an event that is a prerequisite for vesicle clustering (Burré et al., 2010; Burre et al., 2012; Lou et al., 2017). Lou et al. (2017) reported that the facilitation of vesicle docking is abolished when the C-terminal 45 residues of a-Syn, which are required for its interaction with VAMP2, are truncated. Additionally, α-Syn interacts with phosphatidylserine (PS) in *trans* through its amphiphilic N-terminal domain, which facilitates the formation of the SNARE complex, thereby contributing to SNARE-dependent vesicle docking (Lou et al., 2017). It has been reported that α-Syn binds to VAMP2 and the chaperone of the SNARE complex without a concomitant effect on neurotransmission (Burré et al., 2010). However, recent studies have shown that α-Syn and VAMP2 play a cooperative role in SV recycling and the attenuation of neurotransmitter release by limiting SV mobilization and recycling. These results imply that α-Syn/VAMP2 interaction is necessary for α-Syn-induced synaptic attenuation (Sun et al., 2019). Moreover, using synaptic vessel mimics, Diao et al. (2013) demonstrated that vesicle clustering was dependent on the specific interaction of native α-Syn with VAMP2 and anionic lipids. Recently, it was also revealed that, in a mouse model of Parkinson’s disease, environmental enrichment significantly reduced the interaction between pSer129 α-Syn and VAMP2, thereby alleviating non-motor symptoms such as locomotor hyperactivity and anxiety during the early stages of this neurodegenerative condition (Kim et al., 2021). Taken together, these findings indicate that α-Syn/VAMP2 binding is crucial to understanding the pathophysiological function of the former.

### 3.2 SYP and VAMP2: Interacting partners on the SV

One of the most significant roles of SYP is its interaction with VAMP2, which is thought to mediate the targeting of VAMP2 to SVs (Gordon and Cousin, 2014). SYP is the second most abundant protein on SVs (it exists in a 1:2 ratio with VAMP2), and the interaction between these two proteins has been widely investigated (Bacci et al., 2001; Pennuto et al., 2003; Gordon et al., 2011; Kwon and Chapman, 2011; Wilhelm et al., 2014; Kokotos et al., 2019; Wittig et al., 2021). There is evidence to support the suggestion that SYPI plays a role in directing the correct sorting of VAMP2 in neurons. The results of studies in which fluorescent VAMP2 chimeras were expressed in cultured hippocampal neurons indicated that SYPI can regulate the sorting of SV proteins by forming heterodimers with VAMP2; moreover, the effect of SYPI on VAMP2 sorting was found to be dose-dependent and highly specific (Calakos and Scheller, 1994; Washbourne et al., 1995; Pennuto et al., 2002, 2003). It has been proposed that the sole physiologically relevant function of SYP is to coordinate VAMP2 retrieval during SV endocytosis, including its post-fusion trafficking, suggesting that these two proteins have an intricate relationship at the pre-synapse (Cousin, 2021). Chemical cross-linking experiments have also demonstrated that the interaction between SYP with VAMP2 is enhanced after membrane fusion (Khvotchev and Südhof, 2004).

### 3.3 The binding of VAMP2 to SM proteins, the chaperones of SNARE assembly

The modes of SM protein-SNARE interaction have been extensively investigated (Misura et al., 2000; Rizo and Rosenmund, 2008; Diao et al., 2010; Shen et al., 2010, 2018; Yu et al., 2013; Andre et al., 2020). SM proteins are soluble factors with a molecular mass of 60–70 kDa that modulate the speed and help ensure the specificity of vesicle fusion by directly interacting with specific synaptic SNARE complexes (Südhof and Rothman, 2009; Rizo and Südhof, 2012).

The binding of Munc18-1 to the SNARE core is sufficient to stimulate membrane fusion. The binding of SM proteins to v-SNAREs, despite being less well-studied than their binding to t-SNAREs, is conserved among SM proteins (Deak et al., 2009; Lobingier and Merz, 2012; Baker et al., 2015; Zhang et al., 2015; Eisemann et al., 2020). The direct interaction between SM proteins and VAMP2 was not detected in some studies (Latham et al., 2006; Rodkey et al., 2008; Rathore et al., 2019). However, Carpp et al. (2006) demonstrated that a yeast SM protein, Vps45p, can bind to the v-SNARE Snc1p and SYX homolog, indicating that SM proteins may interact with both v-SNAREs and t-SNAREs and thereby establishing a precedent for a putative SM/VAMP2 interaction. In addition, Rodkey et al. (2008) observed detectable, albeit substoichiometric, binding of Munc18a to VAMP2. The authors suggested that Munc18a may actively recruit and position VAMP2 to facilitate SNARE complex formation and, subsequently, membrane fusion. An alternative structural model of SM/v-SNARE interaction has also been proposed. The SM protein Vps33 of yeast binds to the SNARE motifs of the homologous v-SNARE Nyv1 (Xu et al., 2010; Baker et al., 2015; Zhang and Hughson, 2021). In this mode, the SNARE motif of Nyv1 binds to non-overlapping sites of Vps33. Moreover, consistent with a Vps33/Nyv1 binding-like mode, it has been shown that the Munc18-1 hairpin must be in its extended or unfolded conformation for Munc18-1 to be able to bind VAMP2. This suggests that Munc18-1 can serve as a template to bring SYX-1A and VAMP2 into close proximity for the correct assembly of the SNARE complex (Ma et al., 2013; Parisotto et al., 2014; Munch et al., 2016; Wang et al., 2019). A recent study reported that helices 11 and 12 of Munc18-1 together bind the VAMP2 central region (i.e., SNARE motifs) and that this is vital for SNARE complex formation and synaptic transmission (Andre et al., 2020). The authors first identified amino acids involved in Munc18-1/VAMP2 interaction *via* site-specific photo-crosslinking. Then, focusing on glutamine 301 (Q301) in helix 11 of Munc18-1, the authors generated novel loss-of-function (Q301D) and gain-of-function (Q301R) mutants and employed a liposome co-sedimentation assay to measure the binding of VAMP2 and Munc18-1. No increase in small unilamellar vesicle (SUV)/VAMP2 co-sedimentation was detected in the presence of Munc18-1 Q301D, indicating that this mutation impairs the affinity of Munc18-1 for VAMP2. The opposite was observed when Munc18-1 Q301R was employed. Importantly, synaptic transmission and short-term plasticity were assessed in mouse hippocampal neurons harboring the Munc18-1 Q301R and Q301D mutations. The results indicated that mEPSCs and ePESCs were significantly reduced in the synapses of neurons expressing Q301D, indicating that the probability of synaptic transmission and release was greatly reduced with this mutation; in contrast, the recorded mEPSCs and ePESCs of Q301R-expressing synapses were similar to those of wild-type neurons. These experiments demonstrated the physiological significance of Munc18-1/VAMP2 interaction and revealed a critical role for Munc18-1 Q301 in SNARE complex assembly (Jiao et al., 2018; Andre et al., 2020). Moreover, as mentioned below, the VAMP2 JMD is required for SNARE/Munc18-1-mediated membrane fusion (Rathore et al., 2019).

Munc13-1 is another key regulatory factor for SNARE complex formation (Ma et al., 2013; He et al., 2017; Lai et al., 2017; Wang et al., 2017; Shu et al., 2020). Munc13-1 has been shown to stabilize the template complex by interacting with the membrane-proximal linker region of VAMP2 (Sitarska et al., 2017; Wang et al., 2019; Andre et al., 2020). Additionally, Munc13-1 was reported to recruit VAMP2-containing SVs to the target plasma membrane and bring the SNARE motifs of VAMP2 closer to the Munc18-1/SYX-1A complex (Rathore et al., 2019; Wang et al., 2019). This implies that the interaction of VAMP2 with Munc13 is coordinated with that of Munc18-1/Syx-1A to allow the completion of SNARE complex formation (Ma et al., 2013; Yang et al., 2015; He et al., 2017; Wang et al., 2019; Shu et al., 2020; Stepien and Rizo, 2021). Combined, these observations indicate that each of the components required for fusion has multiple mutual affinities, and a host of other fusion components are required to synergize with the SNAREs to achieve the sophisticated regulation of SNARE complex formation (Rizo and Rosenmund, 2008; Südhof and Rothman, 2009; Jahn and Fasshauer, 2012; Sitarska et al., 2017; Jun and Wickner, 2019; Wang et al., 2019; Weiss, 2019). However, the structure of the Munc18-VAMP2 complex needs further investigation at high resolution.

### 3.4 Interaction between VAMP2 and ion channels

VAMP2 interacts with relatively few ion channel proteins. Lvov et al. (2008) were the first to report the interaction between VAMP2 and Kv2.1. In contrast to that seen with t-SNAREs, the Kv2.1-VAMP2 interaction does not involve the C-terminus of Kv2.1. Instead, VAMP2 directly interacts with the T1 domain in the N-terminus of Kv2.1, which enhances the inactivation of the latter. Moreover, Kv2.1-VAMP2 interaction plays a fundamental role in the release of hormones, neuropeptides, and neurotrophic factors from DCVs (Taubenblatt et al., 1999; Fernández-Alfonso et al., 2006).

## 4 The regulation of membrane fusion by VAMP2 domains

Despite the vitally important role of VAMP2 in synaptic fusion events, little is known about the structure and function of VAMP2 before SNARE complex formation (Deák et al., 2006; Wang C. et al., 2020). The results of recent studies have suggested that the structural conformations of the different domains of VAMP2 may determine its various functions in membrane fusion (Chiang et al., 2018).

### 4.1 The regulation of membrane fusion by the TMD

The TMD is mainly composed of hydrophobic residues and can embed into and interact with the vesicle lipid bilayer (Sutton et al., 1998; Han et al., 2004, 2016; Fdez et al., 2010; Fezoua-Boubegtiten et al., 2019; Weiss, 2019). A critical glycine residue (Gly 100) acts as an intrinsic kink in the TMD, which enhances the structural variability of the VAMP2 protein and ensures some flexibility in SNARE complex assembly (Bowen and Brunger, 2006; Han et al., 2016). Dhara et al. (2016) demonstrated that the conformational flexibility of the TMD is pivotal for effective Ca^2+^-triggered vesicle exocytosis and can strongly promote membrane fusion as well as fusion pore opening. Stein et al. (2009) investigated the influence of the TMDs of SNARE complex proteins on the stability of the neuronal SNARE complex by solving the X-ray structure of the latter. The authors reported that, when only the VAMP2 TMD was present, the stability of the SNARE complex was reduced relative to that of the core complex, suggesting that the presence of the C-terminus of VAMP2 hinders the packing of the four-helical bundle further upstream (Stein et al., 2009). However, how the TMD regulates the formation of the SNARE complex remains controversial. Han et al. (2016) used microsecond-long atom molecular dynamics to simulate the insertion of VAMP2 into the membrane aiming to characterize the molecular roles of the TMD sequence on the VAMP2 folding process and the structural and dynamic properties of the other domains of VAMP2. The results showed that the TMD of VAMP2 can regulate the JMD and the linker between the TMD and the JMD, thereby affecting the helicity and flexibility of VAMP2 (Figure 1).

### 4.2 The regulation of membrane fusion by the JMD

The TMD is thought to have a limited effect on the membrane environment. In contrast, the JMD destabilizes the structure and stability of the outer membrane layer, implying that this domain is vital for vesicle fusion (Fdez et al., 2010; Han et al., 2016; Rathore et al., 2019). The JMD motif is more conserved than the TMD motif (Khvotchev and Südhof, 2004) and generally serves as a linker between the TMD and the SNARE motif during membrane fusion (Figure 1; Bowen and Brunger, 2006; Stein et al., 2009; Brewer et al., 2011; Rathore et al., 2019). The JMD also can promote the formation of fusion pores by interacting with other membrane remodeling molecules, such as SYT and the calcium-sensitive double C2-like domain-containing protein beta (DOC2B) (Martens et al., 2007; Lynch et al., 2008; Martens and McMahon, 2008; Hui et al., 2009; Rathore et al., 2019). This implies that the JMD is necessary for membrane stability. Additionally, a robust inhibitory effect on SNARE/Muncl8-1-mediated fusion was observed when the JMD of VAMP2 was mutated (Rathore et al., 2019). Meanwhile, the JMD also acts as an extravesicular regulator of the activities of Munc13 and CPX (Maximov et al., 2009; Fang et al., 2013; DeMill et al., 2014; Scholz et al., 2019; Wang et al., 2019). These observations indicate that the VAMP2 JMD is indispensable for SNARE/Munc18-1-mediated membrane fusion, although no direct structural link has been detected between the JMD of VAMP2 and SNARE/Munc18-1 (Shen et al., 2007; Xu et al., 2010; Yu et al., 2018). Interestingly, studies have suggested that the JMD does not participate in the docking step during the membrane fusion reaction (Yu et al., 2013; Rathore et al., 2019). The docking of t- and v-SNARE liposomes (wild type or mutant) in the presence or absence of Munc18-1 was assessed using a liposome docking assay, with the results showing that liposome docking was not affected by JMD mutation (Rathore et al., 2019).

The JMD can spontaneously fold into a flexible, α-helical conformation, while the TMD retains a rigid helical structure. It was reported that the helix content of the JMD is decreased in VAMP2 mutants compared with that in wild-type VAMP2 and that this difference in helicity implied that the TMD influences the structure of the JMD of VAMP2 (Han et al., 2016). However, the potential mechanism of α-helical conformation in the interaction with membrane has not been fully explained (Bowen and Brunger, 2006; Ellena et al., 2009; Han et al., 2016). Notably, studies have shown that two critical tryptophan residues (Trp89 and Trp90) in the JMD are required to prevent the premature formation of the SNARE complex, thus exerting a regulatory effect on the membrane fusion process (Fulop et al., 2005; Fang et al., 2013). Additionally, substituting these two hydrophobic residues with neutral alanine in the JMD promotes spontaneous membrane fusion and faster transmitter release kinetics (Neher, 2006; Zhang and Jackson, 2010; Fang et al., 2013). In chromaffin cells, the lysine and arginine residues located at the membrane-water interface of the JMD assist in stabilizing the position of the TMD and control the proportion of spontaneous and stimulated membrane fusion events (Bowen and Brunger, 2006; Lindau et al., 2012; Fang et al., 2013). Based on molecular dynamics simulations, the tryptophan residues in the JMD modulate the insertion of portions of the SNARE motif into the bilayer as well as regulate the depth of membrane insertion of peptides. Thus, the potential of the electrostatic surface can be adjusted, and the energy barrier for vesicle fusion can be affected (Bowen and Brunger, 2006; Borisovska et al., 2012; Han et al., 2016). Furthermore, the JMD modulates the availability of VAMP2 by affecting the positioning of SNARE motifs, thus influencing the assembly of the SNARE complex during fusion (Kweon et al., 2003a,b; Han et al., 2016).

### 4.3 The regulation of membrane fusion by other VAMP2 domains

In addition to the TMD and the JMD, numerous studies have shown that VAMP2 controls neurotransmitter release through the interaction of its SNARE motifs with those of other SNAREs. These interactions can facilitate the formation of the SNARE complex and the fusion of SV membranes with the plasma membrane (Brunger et al., 2015, 2018; Rizo, 2018). Nevertheless, SNARE motifs are rich in negatively charged residues and are thus repelled by the negatively charged membrane surface. Accordingly, it is likely that SNARE motifs of most synaptic short peptides cannot bind to membranes but may instead play a necessary role in membrane fusion and its regulation (Brewer et al., 2011). For instance, the interaction between helix 11 and 12 of Munc18-1 with the central region of the VAMP2 SNARE motif is crucial for SNARE templating and synaptic transmission (Andre et al., 2020). Before VAMP2 forms the SNARE complex with its partners, its N-terminal domain is thought to be mainly bound to the C-terminal domain of α-Syn (Hesselbarth and Schmidt, 2021). Given that α-Syn facilitates the assembly of the SNARE complex between VAMP2 and SYX-1A/SNAP-25 dimers at the plasma membrane, this implies that the N-terminus of VAMP2 is not involved in SNARE complex formation (Burre et al., 2012; Gordon and Cousin, 2014; Jumper et al., 2021). In addition, VAMP2 contains a proline-rich domain (PRD) at its N-terminus, which is reported to be involved in SV recycling and VAMP2 retrieval (Wadel et al., 2007; Mori and Takamori, 2017).

## 5 Discussion

Collectively, the VAMP2 protein plays a crucial role both in initiating SNARE complex assembly and promoting vesicle fusion and transmitter release (Diao et al., 2011; Fezoua-Boubegtiten et al., 2019). VAMP2 is an unstructured protein until it interacts with t-SNAREs (Wang C. et al., 2020; Hesselbarth and Schmidt, 2021). Upon binding to membranes or other SNAREs proteins, VAMP2 changes from a naturally unstructured protein to a highly structured component of the SNARE complex. The different domains of VAMP2 perform distinct functions in membrane fusion (Wang C. et al., 2020). The TMD shows strong sensitivity to local protein concentrations and specific lipid environments, while the JMD regulates the function of the TMD. Thus, the two domains jointly regulate the stability of the SNARE complex and vesicular membrane components in different regions.

Although VAMP2 plays a critical role in presynaptic molecular fusion events, the precise molecular mechanisms underlying the regulation of VAMP2 remain unclear and continue to be a major topic of investigation. Moreover, the importance of VMAP2 for synaptic function in mice is evident from the dramatic deficits in synaptic transmission observed in animals lacking VAMP2; however, little is known about the consequences of VAMP2 dysfunction in human neurodevelopment (De Haro et al., 2003; Deák et al., 2006; Chang et al., 2015). Studies have shown that some diseases of nervous system development, including epilepsy and abnormal movement, are caused by VAMP2 gene mutation, which mainly leads to the impairment of presynaptic nerve transmission at nerve terminals (Salpietro et al., 2019). This suggests that VAMP2 mutation may affect synaptic membrane fusion and disrupt human neural development, possibilities that require further extensive investigation.

## Author contributions

CY and WD conceived and designed the study. CY and JJ compiled the figures. All authors drafted and revised the manuscript.
